# Matching Data Splitting to Extrapolation Targets in Soil Machine Learning: When Is Soil-wise Validation Needed?

**DOI:** 10.1021/acs.est.6c06032

**Published:** 2026-07-29

**Authors:** Fengxian Chen, Jan Vanderborght

**Affiliations:** Institute of Bio- and Geosciences, IBG-3 Agrosphere, Forschungszentrum Jülich GmbH, Jülich D-52425, Germany

**Keywords:** machine learning, soil, data splitting, validation

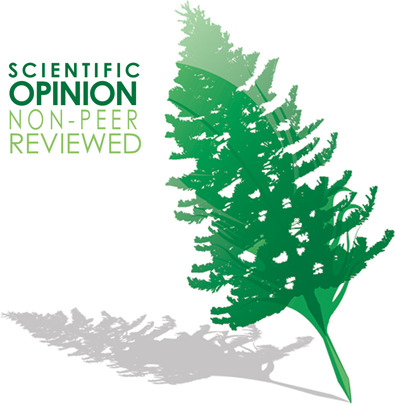

## Graphical abstract

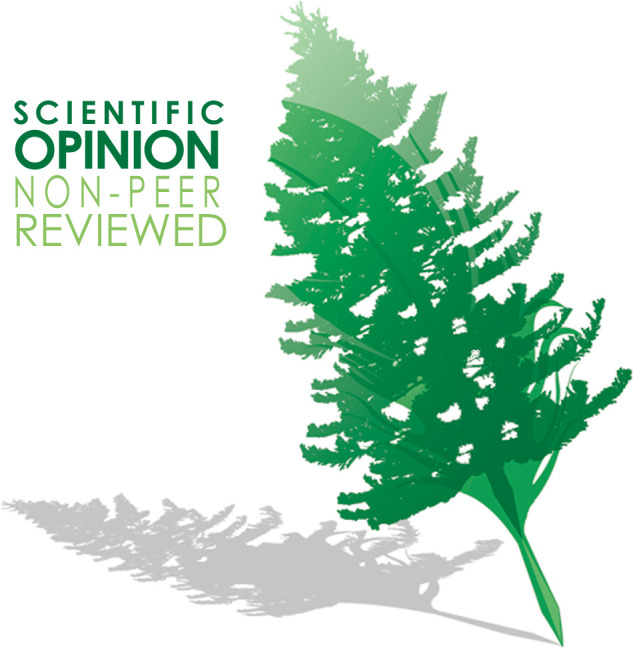

Machine learning (ML) is increasingly used to quantify soil processes, predict environmental risks, and map soil properties at regional to global scales. These applications include estimating transport parameters (e.g., dispersivity), modeling soil–contaminant interactions (e.g., adsorption and degradation), and assessing environmental exposure and contamination risks (e.g., accumulation in plants).^1–4^ Despite their diversity, these tasks share a common objective: to generalize from observed soils to new, unobserved soils. In this Viewpoint, we use “soil” to refer not to an individual measurement or subsample, but to an independent soil unit with a shared pedological, environmental, or experimental context, such as a soil profile, field site, or sampling location.

Most soil data sets are inherently clustered, with multiple observations originating from the same soil under different experimental conditions, depths, or time points. These samples are not independent but share latent characteristics such as mineral composition, structure, and microbial communities. As a result, each soil carries a distinct fingerprint that is only partially captured by measured variables.

As shown in Figure 1, when random data splitting is applied to such clustered data sets, samples from the same soil are often distributed across both training and test sets. This allows ML models to exploit soil-specific information, leading to systematic information leakage. This effect was observed across four representative soil ML tasks involving the prediction of transport, adsorption, degradation, and plant-accumulation parameters. Under random splitting, gradient boosting machine (GBM) models achieved apparently high predictive performance for dispersivity in solute transport models (*R* ^2^ = 0.62 ± 0.04), PFAS adsorption on soil (*R* ^2^ = 0.84 ± 0.02), atrazine degradation in soil (*R* ^2^ = 0.70 ± 0.17), and accumulation of ultraviolet stabilizer benzotriazole in roots (*R* ^2^ = 0.81 ± 0.02). However, when samples from the same soil were kept together using soil-wise splitting, the corresponding performance decreased to −0.34 ± 1.15, 0.77 ± 0.03, 0.25 ± 0.30, and 0.64 ± 0.08, respectively. Details of the data sets and GBM-based calculations are provided in the Supporting Information. Consequently, model performance under random splitting primarily reflects interpolation within known soils rather than true extrapolation to new soils, resulting in inflated *R* ^2^ values and overly optimistic conclusions.

**Figure 1:**
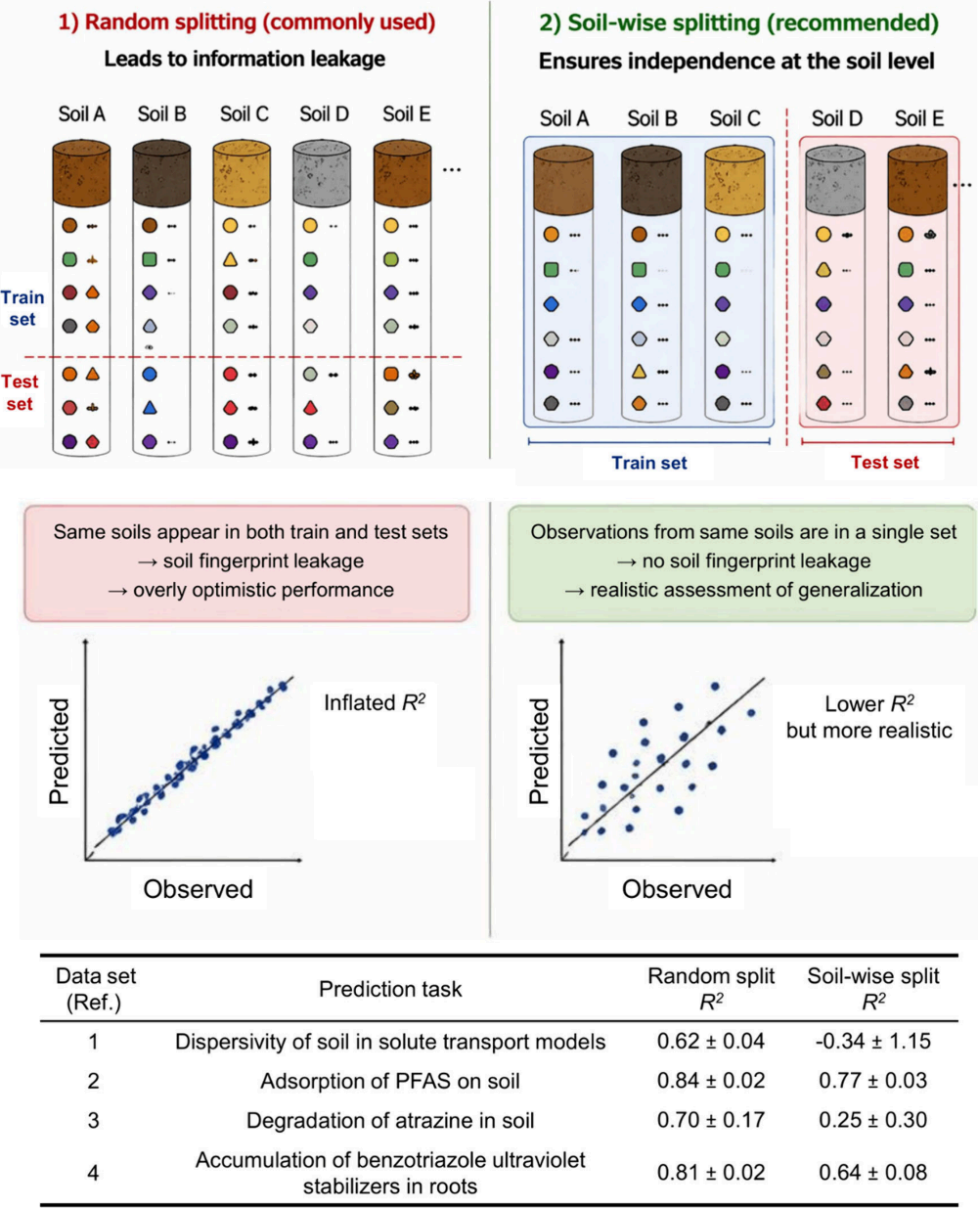
Random vs soil-wise splitting in clustered soil ML data sets. The table summarizes four representative data sets covering dispersivity, PFAS adsorption, atrazine degradation, and accumulation of ultraviolet stabilizer benzotriazole in roots. Part of this figure was adapted from EGU26-19636, originally presented at the EGU General Assembly 2026, under a Creative Commons Attribution 4.0 International License (CC BY 4.0).

Admittedly, soil-wise validation is not universally required for all soil ML studies. Rather, it is required when the intended use of the model is extrapolation to new soils. If the objective is interpolation among repeated measurements from already sampled soils, random splitting may still be informative, but its interpretation should be restricted to within-soil interpolation and should not be used as evidence of transferability to unobserved soils.

Recent studies have proposed a more granular grouping strategy (i.e., soil × compound splitting, data with the same soil and compound are grouped together).^5^ However, it does not correspond to the main extrapolation targets in soil contaminant modeling. If the objective is to predict responses for new soils, independence must be enforced at the soil level; if the objective is to predict responses for new contaminants, independence must be enforced at the compound level. If the objective is to extrapolate simultaneously to new soils and new contaminants, the validation design must ensure that the test set contains soils and contaminants that are both absent from the training set. Soil × compound splitting does not prevent information leakage along the relevant extrapolation dimensions. Although it appears to be more granular, it is misleading because it may give the impression of a stricter validation design while still allowing soil-specific or contaminant-specific information to be shared between training and test sets.

These considerations point to a more general principle. Extrapolation must be defined with respect to a target dimension, and independence must be enforced along that same dimension. We therefore recommend that soil ML studies explicitly state the intended prediction target and choose the validation design accordingly. In most soil ML applications, if the primary objective is to predict outcomes for new soils, soil-wise splitting should be a standard practice.

## Supplementary Material



## Notes

Four data sets used in this study, covering dispersivity in solute transport, PFAS adsorption on soils, atrazine degradation in soils, and the accumulation of ultraviolet stabilizer benzotriazole in roots (for each data set, the input variables, output variable, and soil-group identifier (soil_ID) are provided); code used for data preprocessing, random and soil-wise data splitting, model training, performance evaluation, and prediction; and an overview summarizing the main characteristics of the four data sets (ZIP)
