# Supplementary material for: Matching Data Splitting to Extrapolation Targets in Soil Machine Learning: When Is Soil-wise Validation Needed?
Source: Environ Sci Technol. 2026 Jul 29;60(31):21391–3. doi: 10.1021/acs.est.6c06032 (PMC13472134; doi:10.1021/acs.est.6c06032)
Supplement: Supplementary file 1 [file es6c06032_si_001.zip › Supp/Summary.docx]

| **Case No.** | **Input Variables** | **Target Variable** | **Number of Datasets** | **Number of Soils** | **ML model used in the original study** | **ML model used in this study** | **Reference** |  |
| --- | --- | --- | --- | --- | --- | --- | --- | --- |
| **1** | Sand content, Clay content, Silt content, Bulk density, Water flux | Dispersivity  α | 128 | 24 | Random forest | GBM | Tran et al., 2026.  *Environmental Science & Technology.* |  |
| **2** | Molecular weight, log KOW, Charge density, Soil pH, SOC, CEC  Sand content, Clay content, Silt content | Adsorption  coefficient  K_d_ | 1274 | 426 | K-Nearest Neighbors | GBM | Fabregat-Palau et al., 2025. *Environmental Science & Technology.* |  |
| **3** | Soil pH, SOC, Concentration, Sand content, Clay content, Silt content, Soil moisture, Degradation time | Degradation  coefficient  μ | 327 | 77 | Gradient Boosting Machine | GBM | Chen et al.,2024.  *Journal of Hazardous Materials.* |  |
| **4** | BUV concentration, pH, EC, SOM, CEC, Fe(II) concentration, Fe(III) concentration, Free iron oxide concentration, Exposure period, Molecular weight, lgKOW, lgKOA | Root concentration factor | 309 | 19 | Random forest | GBM | Xu et al., 2025.  *Journal of Hazardous Materials.* | |
